# A Competency Model for Clinical Physicians in China: A Cross-Sectional Survey

**DOI:** 10.1371/journal.pone.0166252

**Published:** 2016-12-09

**Authors:** Zhuang Liu, Lei Tian, Qing Chang, Baozhi Sun, Yuhong Zhao

**Affiliations:** 1 School of Public Health, China Medical University, Shenyang, Liaoning, China; 2 Research Center for Medical Education, China Medical University, Shenyang, Liaoning, China; 3 Department of Clinical Epidemiology, Shengjing Hospital, China Medical University, Shenyang, Liaoning, China; Western Sydney University, AUSTRALIA

## Abstract

**Background:**

Around the world, regulatory bodies have taken the lead in determining the competencies required to become a physician. As a first step in addressing this project, it was decided to develop a set of core competencies that were unique to China and that might serve as a basis for medical education. The purpose of this paper was to construct a competency model for clinical physicians in China.

**Methods:**

Data was collected using a cross-sectional survey of 6247 clinicians from seven administrative regions (31 provinces, autonomous regions and municipalities directly under the central government) in China. The total sample was randomly divided into two sub-samples, an initial sample (Sample 1) and a replication sample (Sample 2). Independent exploratory factor analysis was conducted in each sample and the results were compared to determine the stability. After that the confirmatory factor analysis was used to ascertain the competency model for physicians. The reliability, convergent and discriminant validity of competency-based instrument were also examined.

**Results:**

76 items with 8 dimensions were identified, accounting for 68.41% of the construct’s total variance in the initial sample and 67.47% in the replication sample. For the two samples, the overall scale reliability (Cronbach’s alpha) was both 0.985 with dimensions from 0.905 to 0.954 for the initial sample and from 0.902 to 0.955 for the replication sample after deleting the items. In confirmatory factor analysis, the result showed that all items had acceptable goodness of fit index. RMSEA and SRMR were less than 0.08 (RMSEA = 0.046, SRMR = 0.040), while GFI, NFI, IFI, and CFI were higher than 0.9 (GFI = 0.905, NFI = 0.903, IFI = 0.909, CFI = 0.909), leading to acceptable construct validity. All construct reliability values of the factors were higher than 0.70, and all average variance extracted values exceeded 0.50. Thus, we considered the reliability and validity of the 8 dimensions were acceptable.

**Conclusions:**

The instrument was shown to be both valid and reliable for measuring clinical physicians’ competency in China. The results of the competency-based instrument can be used by ministry of health and administrators of hospitals to assess physicians’ competencies, encourage and guide them to modify their behaviors according to the evaluation criteria, and also cultivate physicians with strong clinical practice, innovation and independent scientific research ability. Through these measurements and understandings, the overall level of clinical physicians will be increased in China.

## Introduction

Over the past 25 years, there has been a significant change in thinking about the way physicians and other professionals are trained. Historically, the emphasis has been on the educational process and the resources available to the students, including the facilities, faculty, content, and length of training. Recently, this focus has begun to shift to outcomes, the competencies a trainee must have on program completion [1, 2]. These competencies or outcomes are then used to decide who should be admitted to the program, the content of the curriculum, the nature of the training sites, and the qualifications of the faculty [3, 4]. Assessments are then targeted to the competencies to determine when individual student achievement is sufficient [5].

Around the world, regulatory bodies have taken the lead in determining the competencies required to become a doctor. The Medical Council of Canada has defined them as the Canadian Medical Education Directives for Specialists (CanMEDS) roles, the General Medical Council (GMC) of the United Kingdom has published them in Good Medical Practice (GMP), and the Accreditation Council for Graduate Medical Education (ACGME) has identified its own set of competencies in the United States [6–9]. These have been very influential both nationally and internationally, despite the fact that there is considerable similarity across them, each set is tailored to the specific needs of the country. The impetus for outcomes-based education increased recently with the publication of the report of the Global Commission on Health Professional Education for the 21st Century [10]. The report suggested that professional education has not kept pace with many of the world’s health care challenges, because of fragmented, outdated, and static curricula that produce ill-equipped graduates. They focused attention on the core competencies required of health care providers, and encouraged renewed commitment to an outcomes-based education model.

In 2008, the China’s Ministry of Health carried out a program that sought to standardize training for resident physicians. At the same time, the Ministry of Education in China prepared a report entitled, “Training Excellent Doctors”. Taken together, these actions led to the question of whether the quality of physicians, and their ability to satisfy the healthcare needs of Chinese people, could be improved by changing the medical education system. As a first step in addressing this question, it was decided to develop a set of core competencies that were unique to China and that might serve as a basis for educational reform. The purpose of this study is to describe the methods used to develop a set of Chinese competencies and to report on the results of this effort.

## Methods

### Form the Questionnaire

In response to the recent call for competency-based educational reform from the Lancet Commission Health professionals for a new century [10], the research team at China Medical University reviewed the international competency literature for clinical physicians as well and the few competencies papers, including the six core competencies adopted by the ACGME, CanMEDS 2005 Physician Competency Framework and GMC (Table 1). Literatures on competencies generated from other disciplines were also consulted [11, 12]. The need to define competencies for physicians in China was recognized and lead to a proposal to the Ministry of Education for support to do a national survey. The preliminary items were generated through a literature review, expert consultation and panel discussion.

**Table 1:** Comparisons of competencies frameworks.

Chinese clinicians’ competencies	ACGME	CanMEDS	GMC
• Information and management	• Medical knowledge	• Communicator	• Medical technique service
• Professionalism	• Patient care	• Collaborator	• Medical diagnosis norm
• Clinical skills and patient care	• Interpersonal communication	• Manager	• Teaching and training skills
• Interpersonal communication	• Professionalism	• Health promoter	• Interpersonal communication
• Health promotion and disease prevention	• Practice-based learning and improvement	• Scholar	• Teamwork
• Master of medical knowledge	• System-based practice	• Professionalism	• Professionalism
• Academic research			
• Teamwork			

First, the research team assembled competency items as listed in the reviewed literature. 350 clinicians (1/3 early in training, 1/3 mid-career, 1/3 senior level) were randomly selected with the help of the personnel office from approximately 3000 physicians across the three affiliated hospitals to receive a paper copy of the instrument. They were asked to rate the importance of the competency items on a 5-Likert scale (not important to very important). From the process above, the first version of the competency model was generated yielding a first draft questionnaire of 69 items.

Secondly, a one-day conference was held with two leaders from each of 31 medical schools (27 provinces plus 4 schools from big cities) that were non-randomly selected based on desired attributes of institutions regarding student mix and institution type as well as working knowledge of education leaders within those institutions. Leadership from the Ministry of Education and Public Health participated but didn’t vote on process that resulted in the final instrument. Subsequent discussion was followed by 60 of the leadership from schools completing the paper survey and their suggestions were used to refine the instrument further. In brief, they thought the 69 items needed to be expanded further. The conference concluded with a discussion on the methodology by which a national survey could be completed utilizing the participating schools.

After the conference, we constructed the competency model with 117 items. Then, we consulted to 10 medical experts who were invited to assign topics for the medical licensing examination every year with the support from National Medical Examination Center of China. They expressed their opinions about the competencies that physicians should have. According to their answers, we revised the content of the questionnaire to suit the culture and conditions of China. After that we invited 60 experts from medical education, clinical medicine, social medicine, statistical and epidemiology specialty, to score the competencies on a 5-point Likert-scale, rank the categories’ importance from 1 (definitely not important), 2 (not important), 3 (neural), 4 (important) to 5 (definitely important). According to expert opinions and practices of knowledge, the competency model was classified to 8 categories and 103 items.

### Subjects and Settings

The study protocol was approved by the China Medical University Review Board. The questionnaire excluded any identifying information about the individual subjects. The purpose of the study was explained to the subjects completely and clearly. Each participant gave verbal and written consent. Participants could retreat from the study at any time and had the right to omit questions if they did not wish to answer.

The method of stratified sampling was used to ensure representation from the different regions. The survey was conducted in seven administrative regions (31 provinces, autonomous regions and municipalities directly under the central government) of China from October 2012 to June 2013 [13], using a cross-sectional study design. The surveyors received training to ensure they understood the questionnaire absolutely. The responses of the participants were anonymous.

### Data Collection

Each province is treated as an investigation unit. Investigation in each unit is organized by the department of teaching affairs of medical school in that province. Before starting the survey, investigators were trained, and funds were allocated for the payment of respondents. Investigation units are responsible for the contact with the surveyed medical institutions in their provinces to deliver and collect of questionnaires and interviews. Questionnaires and the recordings of interviews were mailed back to us.

### Data Analysis

We conducted replication exploratory factor analysis (EFA) by principal component with promax oblique rotation to explore the structure of the instrument and extract important factors [14]. The promax rotation allowed correlation between the factors [15, 16]. And confirmatory factor analysis (CFA) by max likelihood method was used to determine the factor structure of our questionnaire [17]. In view of the large number of items we adopted a cautious strategy and divided the factor analysis into several consecutive steps. Firstly, we used Kaiser Meyer Olkin (KMO) test and Bartlett’s test of sphericity to identify sampling adequacy for factor analysis [18]. If the value of KMO is greater than 0.5 and the significance level of Bartlett’s test of sphericity was less than 0.05, the sample is considered to be suitable for factor analysis. Secondly, we randomly divided the dataset into two parts. Independent EFA was conducted in each sample and results were compared with each of the individual items which led to the division of the scale. Items were removed with loadings on non-concordant factors, or the squared differences achieve a magnitude of 0.04 in the factor loadings. Thirdly, we validated the result of the newly constituted scale with CFA using the full sample [19]. Also, the degree of stability of the results obtained before can be verified. The model fit was assessed by several indices: the goodness of fit index (GFI), the normal fit index (NFI), the incremental fit index (IFI), the comparative fit index (CFI), the root mean square error of approximation (RMSEA), and the standardized root mean square residual (SRMR) [20]. The GFI, NFI, IFI, and CFI values higher than 0.90, the RMSEA values less than 0.10, and SRMR values less than 0.08 indicated acceptable model fit [21–23]. To estimate the reliability of the measurements, we calculated Cronbach’s alpha for all main factors and for each dimension [24]. An alpha of 0.7 to 0.9 was considered to represent an acceptable degree of internal consistency. For the validity of the measurements, the values of construct reliability (CR) and the average variance extracted (AVE) were calculated [18].

Double data entry was performed using Epidata software to ensure the accuracy of the data. Data was analyzed using SPSS version 19.0 (SPSS Inc., Chicago, IL, USA) for EFA and AMOS version 20.0 for CFA. Internal consistency was assessed by calculating the Cronbach’s alpha coefficient.

## Results

### Demographical Characteristics

Among the total of 7019 questionnaires distributed in the formal survey, 6247 valid questionnaires (89%) were collected. The average age of the clinical physicians was 38.98 years (SD = 8.71), with 3523 male clinicians (54.6%) and 2724 female clinicians (43.6%). The demographic characteristics of the clinicians were shown in Table 2.

**Table 2:** Demographic characteristics.

Characteristic		N (%)
Gender	Male	3523 (54.5%)
	Female	2724 (43.6%)
Region	Northern China	1128 (18.1%)
	Northeast China	661 (10.6%)
	Eastern China	1146 (18.3%)
	Central China	706 (11.3%)
	Southern China	466 (7.5%)
	Southwest China	1017 (16.3%)
	Northwest China	1123 (18.0%)
Academic degree	Bachelor	3030 (48.5%)
	Master	2386 (38.2%)
	Doctor	831 (13.3%)
Title	Primary	1574 (25.2%)
	Middle	1693 (27.1%)
	Vice-senior	1768 (28.3%)
	Senior	1212 (19.4%)
Work experience in equivalent-years	Below 5 years	1293 (20.7%)
	5 to 9 years	1056 (16.9%)
	10 to 14 years	1006 (16.1%)
	15 to 19 years	1106 (17.7%)
	20 years and above	1786 (28.6%)

### Exploratory Factor Analysis

The Cronbach’s alpha was applied to estimate the scale reliability, and the Cronbach’s alpha was 0.989, demonstrating strong internal consistency. The KMO value was 0.991 and Bartlett’s spherical test was found to be statistically significant (P<0.001). As a result, the study data were suitable for factor analysis.

All the competency questionnaires answered by clinical physicians were randomly divided into two parts, and were analyzed with independent EFA. During the attempt of EFA, items were removed with loading on non-concordant factors, or the squared differences achieve a magnitude of 0.04 in the factor loadings. For example, Item 1.14 had the highest factor loading on Factor III in Sample 1 and on Factor V in Sample 2. This item is probably not a good one, and would benefit from deletion. The factor loadings of each item for the two samples were represented in Tables 3 and 4. Similarly, we removed 27 items until all the items satisfied the item inclusion and exclusion criteria across both samples. (Table 5) For Sample 1, the overall scale Cronbach’s alpha was 0.985 with dimensions Cronbach’s alpha from 0.905 to 0.954 after deleting the items. For Sample 2, the overall scale Cronbach’s alpha was 0.985 with dimensions Cronbach’s alpha from 0.902 to 0.955. Finally, 76 items with 8 dimensions were identified, accounting for 68.41% of the construct’s total variance in the initial sample and 67.47% in the replication sample (Table 5), which meeting Hair et al.’s criterion [18].

**Table 3:** The factor loadings after promax rotation in Sample 1.

Items	Communality	Factor Loadings							
	Extract	1	2	3	4	5	6	7	8
1.1	0.584	0.249	0.509	**0.707**	0.406	0.326	0.439	0.208	0.334
1.2	0.646	0.303	0.500	**0.769**	0.415	0.360	0.432	0.269	0.310
1.3	0.641	0.335	0.497	**0.781**	0.445	0.387	0.432	0.359	0.282
1.4	0.610	0.427	0.515	**0.777**	0.494	0.437	0.427	0.450	0.141
1.5	0.627	0.339	0.526	**0.781**	0.439	0.376	0.438	0.329	0.207
1.6	0.659	0.399	0.542	**0.810**	0.485	0.449	0.447	0.419	0.100
1.7	0.652	0.363	0.530	**0.804**	0.476	0.431	0.429	0.379	0.150
1.8	0.634	0.412	0.545	**0.795**	0.492	0.456	0.460	0.396	0.159
1.9	0.620	0.453	0.517	**0.775**	0.464	0.479	0.400	0.477	0.046
1.10	0.589	0.451	0.531	**0.755**	0.480	0.515	0.431	0.481	0.077
1.11	0.587	0.523	0.438	**0.559**	0.452	0.494	0.343	0.509	-0.115
1.12	0.570	0.523	0.469	**0.577**	0.510	0.488	0.372	0.569	-0.076
1.13	0.569	0.479	0.561	**0.633**	0.548	0.496	0.452	0.461	0.060
1.14	0.514	0.500	0.605	**0.632**	0.530	0.570	0.455	0.579	0.163
1.15	0.528	0.341	**0.639**	0.615	0.481	0.445	0.472	0.303	0.385
1.16	0.550	0.547	0.636	0.599	0.553	0.607	0.456	**0.637**	0.119
1.17	0.552	0.422	0.608	**0.640**	0.548	0.486	0.525	0.379	0.291
1.18	0.516	0.493	0.640	0.622	**0.660**	0.561	0.474	0.511	0.220
1.19	0.548	0.555	0.545	0.598	**0.651**	0.573	0.434	0.625	0.116
2.1	0.628	0.017	**0.762**	0.073	-0.103	-0.001	0.056	-0.024	0.059
2.2	0.653	0.007	**0.789**	0.040	-0.080	-0.055	0.047	0.080	0.012
2.3	0.670	-0.034	**0.813**	0.044	-0.045	-0.081	0.161	-0.145	0.104
2.4	0.700	-0.036	**0.872**	-0.001	-0.034	-0.030	0.045	0.007	-0.011
2.5	0.709	0.051	**0.873**	-0.028	-0.033	-0.047	0.082	-0.062	-0.018
2.6	0.661	0.053	**0.798**	-0.062	-0.022	-0.027	-0.036	0.144	-0.038
2.7	0.660	0.046	**0.737**	-0.051	0.022	0.007	-0.049	0.170	-0.085
2.8	0.682	0.005	**0.823**	-0.094	0.018	0.013	-0.049	0.135	-0.055
2.9	0.629	-0.035	**0.765**	-0.012	0.051	-0.020	0.127	-0.098	0.007
2.10	0.639	0.028	**0.754**	-0.016	0.045	0.026	0.064	-0.107	0.029
2.11	0.580	0.015	**0.672**	-0.015	0.038	0.009	0.014	0.099	-0.048
2.12	0.656	-0.006	**0.705**	-0.036	0.044	0.056	-0.028	0.142	-0.043
2.13	0.615	0.060	**0.531**	-0.020	0.085	0.063	-0.126	0.280	-0.028
2.14	0.564	-0.066	0.109	0.089	0.166	0.192	-0.033	**0.287**	0.280
2.15	0.581	-0.066	0.163	0.120	0.253	0.159	-0.036	0.140	**0.290**
2.16	0.684	-0.042	0.082	-0.035	0.093	0.026	-0.151	**0.800**	0.168
2.17	0.649	-0.035	0.071	-0.044	0.125	-0.081	-0.081	**0.793**	0.214
2.18	0.691	-0.020	0.040	-0.094	0.128	-0.035	-0.100	**0.840**	0.177
2.19	0.620	0.023	0.060	0.008	0.307	-0.020	-0.061	**0.474**	0.240
2.20	0.622	0.000	0.173	-0.057	0.184	0.016	-0.087	**0.583**	0.145
2.21	0.676	0.028	0.046	-0.029	0.179	0.059	-0.100	**0.661**	0.112
3.1	0.639	0.032	0.039	0.047	**0.618**	-0.006	0.037	0.097	0.059
3.2	0.675	-0.010	0.029	0.014	**0.726**	0.002	0.063	-0.001	0.090
3.3	0.720	-0.046	0.026	-0.007	**0.779**	0.040	0.037	0.060	-0.021
3.4	0.699	-0.044	0.066	-0.048	**0.816**	-0.017	0.087	-0.014	-0.020
3.5	0.700	0.005	0.045	-0.029	**0.773**	0.008	0.080	-0.006	-0.023
3.6	0.715	0.035	0.010	-0.008	**0.732**	0.067	-0.034	0.103	-0.032
3.7	0.702	0.033	-0.065	0.004	**0.819**	-0.046	0.028	0.098	-0.036
3.8	0.725	0.085	-0.003	0.000	**0.847**	-0.086	0.100	-0.093	0.014
3.9	0.710	0.038	-0.090	0.027	**0.798**	0.043	0.052	0.023	-0.044
3.10	0.678	0.079	-0.050	0.028	**0.697**	-0.028	-0.014	0.178	-0.015
3.11	0.645	0.090	-0.066	0.031	**0.656**	-0.032	0.078	0.132	-0.022
3.12	0.627	0.044	-0.046	0.039	0.075	0.113	0.160	**0.516**	-0.001
3.13	0.637	0.088	-0.117	0.001	-0.025	0.009	0.218	**0.668**	-0.003
4.1	0.676	0.115	-0.071	-0.028	-0.145	-0.050	0.348	**0.687**	-0.087
4.2	0.644	0.082	0.019	-0.014	-0.114	-0.088	**0.546**	0.468	-0.079
4.3	0.631	-0.015	0.043	0.070	0.065	-0.117	**0.781**	-0.053	0.047
4.4	0.648	-0.013	0.043	0.018	-0.074	0.042	**0.625**	0.282	-0.142
4.5	0.713	0.073	-0.082	-0.005	-0.065	0.016	0.417	**0.578**	-0.150
4.6	0.678	0.078	-0.009	-0.024	-0.011	-0.043	**0.516**	0.439	-0.103
4.7	0.670	0.042	0.089	0.020	0.108	-0.059	**0.751**	-0.121	0.018
4.8	0.660	0.049	0.086	0.018	0.034	-0.080	**0.720**	0.057	-0.017
4.9	0.703	0.048	0.147	-0.013	0.085	-0.025	**0.744**	-0.117	0.000
4.10	0.664	0.006	0.070	0.016	0.112	0.093	**0.646**	-0.051	-0.012
4.11	0.659	0.077	0.015	-0.006	0.062	0.076	**0.581**	0.115	0.004
5.1	0.656	-0.015	-0.016	0.013	0.058	0.158	**0.591**	0.076	0.095
5.2	0.657	0.048	-0.006	0.029	0.016	0.193	**0.542**	0.068	0.073
5.3	0.639	-0.039	-0.024	0.012	0.044	0.216	**0.550**	0.106	0.076
5.4	0.618	0.017	-0.007	0.024	0.019	0.258	**0.471**	0.082	0.081
5.5	0.684	0.030	-0.034	0.020	-0.027	0.240	0.134	0.092	**0.591**
5.6	0.699	0.018	0.004	-0.024	-0.055	0.182	0.122	0.136	**0.631**
5.7	0.703	-0.010	-0.074	0.008	-0.026	0.191	0.041	0.178	**0.690**
5.8	0.692	0.028	-0.052	-0.015	0.023	0.205	0.065	0.019	**0.718**
5.9	0.690	-0.022	-0.016	-0.012	0.051	0.167	0.115	-0.028	**0.718**
5.10	0.688	0.061	-0.042	-0.030	-0.049	0.074	0.019	0.176	**0.693**
6.1	0.627	0.013	0.027	0.026	0.061	**0.749**	0.135	-0.199	-0.014
6.2	0.683	0.110	0.021	-0.003	0.065	**0.818**	-0.005	-0.168	-0.067
6.3	0.734	0.145	0.026	0.000	-0.002	**0.774**	-0.054	-0.001	-0.098
6.4	0.760	0.122	0.010	0.013	-0.020	**0.794**	-0.054	0.033	-0.087
6.5	0.742	0.153	0.022	-0.006	0.012	**0.804**	-0.006	-0.099	-0.041
6.6	0.752	0.154	0.005	-0.010	-0.038	**0.773**	-0.029	0.035	-0.087
6.7	0.757	0.184	-0.015	-0.037	-0.020	**0.760**	-0.060	0.067	-0.086
6.8	0.732	0.261	-0.022	-0.015	-0.016	**0.681**	-0.019	0.015	-0.051
6.9	0.672	0.335	0.001	0.002	-0.110	**0.348**	0.012	0.285	0.037
6.10	0.699	**0.396**	-0.010	-0.023	-0.111	0.302	-0.060	0.364	0.012
7.1	0.609	**0.639**	0.000	-0.012	-0.002	0.081	0.044	0.063	0.096
7.2	0.645	**0.664**	0.024	0.007	-0.010	0.085	0.055	0.026	0.069
7.3	0.617	**0.625**	0.073	-0.007	0.057	0.110	0.015	-0.026	0.044
7.4	0.567	**0.573**	0.123	0.021	0.127	0.077	0.211	-0.410	0.140
7.5	0.633	**0.687**	0.077	0.003	0.096	0.126	0.047	-0.211	0.079
7.6	0.663	**0.762**	0.070	0.007	0.058	0.057	-0.005	-0.106	0.054
7.7	0.692	**0.736**	0.047	-0.001	0.086	0.204	-0.075	-0.145	0.024
7.8	0.704	**0.766**	0.031	-0.050	0.104	0.177	-0.068	-0.117	0.023
7.9	0.684	**0.713**	0.002	-0.035	0.003	0.097	-0.056	0.115	0.012
7.10	0.599	**0.844**	0.009	0.027	0.004	-0.109	0.019	-0.052	0.139
7.11	0.607	**0.769**	-0.010	0.001	0.049	-0.063	0.097	-0.053	0.078
7.12	0.695	**0.805**	0.011	0.001	0.034	0.088	-0.054	-0.051	0.066
7.13	0.687	**0.750**	0.024	-0.032	0.025	0.059	-0.004	0.029	0.051
7.14	0.633	**0.544**	-0.058	0.034	-0.063	0.092	-0.053	0.279	0.263
8.1	0.644	0.267	-0.099	0.007	-0.027	-0.051	0.008	**0.629**	0.341
8.2	0.685	0.108	-0.042	0.000	-0.019	-0.094	0.023	**0.707**	0.467
8.3	0.696	0.174	-0.075	-0.004	-0.034	-0.150	0.057	**0.694**	0.507
8.4	0.700	0.131	-0.028	0.009	-0.086	-0.124	0.030	**0.747**	0.477
8.5	0.652	0.181	-0.009	0.021	-0.122	-0.111	0.015	**0.694**	0.453

**Table 4:** The factor loadings after promax rotation in Sample 2.

Items	Communality	Factor Loadings								Squared Diff
	Extract	1	2	3	4	5	6	7	8	
1.1	0.566	-0.027	0.079	**0.722**	-0.119	-0.108	0.054	0.009	0.272	0.0002
1.2	0.635	-0.020	-0.030	**0.793**	-0.036	-0.119	0.067	0.028	0.249	0.0005
1.3	0.626	-0.018	-0.005	**0.806**	-0.036	-0.086	0.082	-0.007	0.145	0.0006
1.4	0.606	0.046	-0.032	**0.730**	0.006	0.114	0.005	-0.051	-0.007	0.0022
1.5	0.652	-0.017	-0.038	**0.823**	0.009	-0.099	0.012	0.058	0.128	0.0017
1.6	0.653	0.034	-0.011	**0.790**	0.035	-0.017	-0.027	0.016	-0.009	0.0004
1.7	0.670	-0.021	0.035	**0.805**	0.001	-0.052	-0.038	0.078	0.004	0.0000
1.8	0.646	-0.041	0.028	**0.802**	0.049	-0.075	0.031	0.007	-0.087	0.0000
1.9	0.655	0.027	-0.057	**0.784**	0.046	0.034	0.036	-0.041	-0.169	0.0000
1.10	0.612	-0.031	0.023	**0.705**	-0.014	0.082	0.032	0.034	-0.149	0.0025
1.11	0.620	0.053	0.004	**0.497**	0.036	0.404	-0.064	-0.140	-0.297	0.0038
1.12	0.629	0.010	-0.043	**0.574**	0.124	0.299	-0.119	-0.026	-0.295	0.0000
1.13	0.575	0.030	0.049	**0.622**	0.160	-0.024	-0.046	0.028	-0.180	0.0001
1.14	0.517	-0.026	0.291	0.284	-0.086	**0.403**	-0.041	-0.026	0.083	Failed
1.15	0.538	0.068	**0.526**	0.236	-0.135	-0.029	-0.104	0.094	0.270	Failed
1.16	0.585	-0.040	0.432	0.154	-0.107	**0.449**	0.022	-0.069	-0.006	Failed
1.17	0.533	0.084	**0.598**	0.186	-0.096	-0.035	-0.114	0.103	0.119	Failed
1.18	0.531	0.039	**0.502**	0.192	-0.058	0.151	-0.038	0.018	0.051	Failed
1.19	0.592	0.063	**0.491**	0.108	-0.087	0.342	0.054	-0.121	-0.031	Failed
2.1	0.608	-0.037	**0.776**	-0.013	0.009	-0.099	0.083	0.037	0.084	0.0001
2.2	0.627	0.000	**0.831**	-0.034	-0.068	0.016	0.046	-0.024	0.050	0.0017
2.3	0.670	-0.009	**0.830**	0.052	-0.016	-0.229	-0.053	0.121	0.151	0.0002
2.4	0.703	-0.035	**0.878**	-0.041	0.032	-0.119	0.069	0.023	-0.004	0.0000
2.5	0.677	0.000	**0.857**	-0.046	0.013	-0.100	0.070	0.008	0.000	0.0002
2.6	0.663	-0.009	**0.775**	-0.099	0.084	0.079	0.038	-0.040	-0.075	0.0005
2.7	0.645	0.017	**0.673**	-0.078	0.095	0.131	0.048	-0.030	-0.056	0.0040
2.8	0.652	-0.033	**0.767**	-0.032	-0.019	0.153	0.019	-0.022	-0.041	0.0031
2.9	0.594	-0.012	**0.712**	0.012	0.085	-0.098	-0.011	0.092	0.018	0.0028
2.10	0.622	0.023	**0.732**	-0.013	0.050	-0.070	-0.033	0.106	0.046	0.0004
2.11	0.547	0.042	**0.578**	-0.012	0.049	0.154	0.010	0.014	0.021	0.0088
2.12	0.625	0.023	**0.612**	-0.022	0.087	0.165	0.024	-0.027	0.006	0.0086
2.13	0.614	0.036	**0.453**	-0.050	0.099	0.419	-0.009	-0.099	-0.026	0.0060
2.14	0.600	0.012	0.152	0.003	0.175	**0.491**	0.027	-0.079	0.305	Failed
2.15	0.582	0.082	0.129	0.031	0.247	**0.320**	-0.039	0.005	0.327	Failed
2.16	0.680	-0.057	0.042	-0.053	0.135	**0.774**	0.083	-0.116	0.116	Failed
2.17	0.649	0.007	-0.038	-0.051	0.165	**0.771**	-0.012	-0.065	0.169	Failed
2.18	0.667	-0.063	-0.016	-0.065	0.122	**0.836**	0.056	-0.104	0.114	Failed
2.19	0.607	0.043	0.066	0.003	0.302	**0.451**	-0.050	0.015	0.205	Failed
2.20	0.607	0.037	0.079	-0.022	0.264	**0.522**	0.021	-0.063	0.091	Failed
2.21	0.646	0.035	-0.002	-0.033	0.190	**0.670**	0.035	-0.069	0.102	Failed
3.1	0.626	0.005	0.034	0.016	**0.659**	0.065	0.018	0.038	0.080	0.0016
3.2	0.689	0.018	-0.001	0.006	**0.814**	-0.019	-0.069	0.067	0.081	0.0077
3.3	0.720	-0.027	0.029	-0.009	**0.828**	-0.012	0.030	0.018	-0.005	0.0024
3.4	0.693	-0.036	0.096	-0.029	**0.773**	-0.054	0.062	0.034	0.021	0.0018
3.5	0.675	-0.007	0.064	0.015	**0.750**	-0.011	0.064	-0.018	-0.020	0.0005
3.6	0.689	0.023	0.024	0.009	**0.708**	0.086	0.024	0.013	-0.040	0.0005
3.7	0.684	0.060	0.022	0.015	**0.735**	0.029	-0.022	0.036	-0.021	0.0070
3.8	0.686	0.023	0.029	0.039	**0.785**	-0.069	-0.049	0.086	0.030	0.0038
3.9	0.668	0.092	-0.067	0.026	**0.722**	0.053	-0.034	0.075	0.001	0.0057
3.10	0.662	0.084	-0.040	0.029	**0.661**	0.180	-0.055	0.016	-0.053	0.0012
3.11	0.641	0.054	-0.047	0.009	**0.668**	0.156	-0.057	0.075	-0.078	0.0001
3.12	0.623	0.002	-0.070	0.005	0.124	**0.627**	0.077	0.083	-0.009	Failed
3.13	0.651	0.119	-0.103	-0.065	-0.028	**0.752**	-0.003	0.123	-0.044	Failed
4.1	0.671	0.153	-0.115	-0.038	-0.200	**0.746**	-0.019	0.248	-0.137	Failed
4.2	0.628	0.180	-0.026	-0.040	-0.171	**0.529**	-0.084	0.441	-0.082	Failed
4.3	0.598	0.032	0.111	0.005	0.017	0.042	-0.131	**0.687**	0.134	Failed
4.4	0.642	0.055	0.042	-0.032	-0.136	0.323	0.009	**0.607**	-0.081	Failed
4.5	0.685	0.042	-0.064	-0.087	-0.095	**0.632**	0.018	0.397	-0.142	Failed
4.6	0.647	0.100	-0.023	-0.059	-0.060	0.006	**0.488**	0.422	-0.090	0.0007
4.7	0.650	-0.001	0.056	0.036	0.044	0.004	**0.719**	-0.045	0.128	0.0010
4.8	0.637	0.043	0.017	0.075	0.045	0.050	**0.657**	-0.014	0.015	0.0039
4.9	0.674	0.051	0.020	0.033	0.076	0.080	**0.745**	0.000	0.048	0.0000
4.10	0.662	0.031	0.051	0.031	0.078	-0.062	**0.668**	0.085	0.026	0.0004
4.11	0.647	0.005	-0.010	0.045	0.035	0.128	**0.598**	0.110	-0.041	0.0002
5.1	0.660	-0.024	0.049	0.000	0.136	0.004	0.125	**0.615**	-0.014	Failed
5.2	0.669	-0.057	0.032	-0.008	0.132	0.032	0.174	**0.612**	-0.032	Failed
5.3	0.659	-0.010	0.089	-0.024	0.090	0.006	0.195	**0.570**	-0.018	Failed
5.4	0.647	-0.087	0.048	0.030	0.101	0.042	0.246	**0.540**	-0.021	Failed
5.5	0.648	0.015	-0.064	0.060	0.023	0.268	0.211	0.100	**0.514**	0.0059
5.6	0.672	0.038	-0.058	0.029	0.040	0.241	0.215	0.008	**0.584**	0.0022
5.7	0.705	0.040	-0.096	0.047	-0.013	0.213	0.277	0.037	**0.591**	0.0098
5.8	0.691	0.069	-0.059	0.077	0.024	0.261	0.084	0.050	**0.627**	0.0082
5.9	0.663	0.055	-0.005	0.064	0.054	0.241	0.014	0.084	**0.614**	0.0108
5.10	0.687	0.094	-0.056	0.050	-0.061	0.161	0.228	0.008	**0.611**	0.0067
6.1	0.626	0.053	0.100	0.021	0.008	**0.741**	-0.219	0.112	0.117	0.0000
6.2	0.659	0.122	0.088	0.008	-0.027	**0.735**	-0.149	0.074	0.034	0.0068
6.3	0.734	0.114	0.035	-0.004	-0.007	**0.744**	-0.008	0.014	-0.078	0.0009
6.4	0.737	0.085	0.014	-0.022	-0.040	**0.772**	0.036	0.034	-0.030	0.0004
6.5	0.721	0.140	0.097	-0.033	-0.005	**0.709**	-0.051	0.039	-0.057	0.0090
6.6	0.738	0.148	-0.024	0.014	-0.013	**0.704**	0.068	-0.006	-0.072	0.0047
6.7	0.754	0.131	0.017	-0.012	-0.022	**0.729**	0.097	-0.058	-0.083	0.0009
6.8	0.723	0.185	0.048	-0.028	0.059	**0.677**	-0.006	-0.052	-0.095	0.0000
6.9	0.678	0.368	-0.028	0.012	-0.104	0.301	**0.382**	-0.069	0.023	Failed
6.10	0.690	0.323	-0.062	-0.002	-0.108	0.319	**0.449**	-0.075	-0.034	Failed
7.1	0.645	**0.681**	-0.023	0.016	-0.031	0.041	0.145	0.006	0.004	0.0017
7.2	0.667	**0.681**	-0.021	0.011	-0.006	-0.002	0.195	-0.011	-0.023	0.0002
7.3	0.630	**0.611**	0.042	0.032	0.072	-0.112	0.236	-0.027	-0.041	0.0001
7.4	0.556	**0.666**	0.125	0.070	0.147	-0.406	0.067	0.073	0.065	0.0086
7.5	0.644	**0.739**	0.085	-0.027	0.034	-0.072	0.074	-0.003	0.004	0.0027
7.6	0.673	**0.740**	0.029	-0.003	0.032	-0.084	0.191	-0.072	-0.041	0.0004
7.7	0.671	**0.747**	0.035	-0.001	0.034	-0.058	0.152	-0.071	-0.025	0.0001
7.8	0.688	**0.726**	0.034	-0.035	0.056	-0.046	0.189	-0.080	-0.056	0.0016
7.9	0.690	**0.719**	-0.007	-0.055	0.009	0.149	0.116	-0.120	-0.111	0.0000
7.10	0.611	**0.833**	-0.070	0.000	0.033	-0.034	-0.027	0.017	-0.038	0.0001
7.11	0.629	**0.800**	0.004	-0.021	0.064	-0.045	0.007	-0.020	-0.082	0.0009
7.12	0.675	**0.744**	-0.038	-0.025	0.034	0.030	0.104	-0.017	-0.041	0.0037
7.13	0.674	**0.735**	-0.034	-0.019	0.041	0.078	0.074	-0.042	-0.061	0.0002
7.14	0.619	**0.638**	-0.034	0.045	-0.087	0.236	0.022	-0.049	0.139	0.0088
8.1	0.649	0.038	-0.044	0.008	-0.026	0.212	-0.109	**0.729**	0.096	0.0100
8.2	0.687	0.107	0.030	0.000	-0.022	0.044	-0.165	**0.801**	0.140	0.0000
8.3	0.649	0.133	0.039	-0.013	-0.004	0.049	-0.198	**0.778**	0.148	0.0022
8.4	0.682	0.109	0.025	0.021	-0.019	-0.003	-0.125	**0.810**	0.127	0.0003
8.5	0.644	0.095	0.018	0.008	-0.004	0.028	-0.203	**0.762**	0.092	0.0046

**Table 5:** The cumulative variance and Cronbach’s alpha in two samples after deleting items.

	Sample 1 (n = 3105)		Sample 2 (n = 3142)	
	Cumulative Variance (%)	Cronbach’s alpha	Cumulative Variance (%)	Cronbach’s alpha
**Factor I: Information and management**	47.69	0.954	46.72	0.955
7.1 Search and analyze medical information from different databases				
7.2 Use information technology efficiently to help the diagnosis and patient education				
7.3 Reasonable control of the patient's medical expenses				
7.4 Maintain complete medical record				
7.5 Effectively plan the work and career				
7.6 Appropriate use of time, plan to handle own activities				
7.7 Management capabilities, including patient management, internship student management				
7.8 Constantly improve management capacities of organization and coordination in practice				
7.9 Adequately demonstrate leadership in the team				
7.10 Master at least one foreign language				
7.11 Computer skills				
7.12 Provide guidance and teaching to colleague and medical students if necessary				
7.13 Apply the knowledge and technology of pedagogic to faculty training				
7.14 Evaluate the training object using advanced clinical assessment				
**Factor II: Professionalism**	54.32	0.947	53.35	0.942
2.1 Adhere to heal the sick, serving the people wholeheartedly				
2.2 Love one’s own career				
2.3 Responsibility				
2.4 Sincere and trustworthy				
2.5 Self-regulation				
2.6 Sympathy to patients				
2.7 Patients first, maintaining patients' rights and interests				
2.8 Fair and honest				
2.9 Self protection awareness with legal				
2.10 Precise and careful				
2.11 Doctors should pay attention to their health				
2.12 Patience and endurance				
2.13 Emphasis on self-evaluation and peer-review				
**Factor III: Clinical skills and patient care**	57.79	0.931	57.07	0.929
1.1 Prudent practice, pay attention to patients' safety				
1.2 Collect important medical histories				
1.3 Complete medical documents according to specifications				
1.4 Understand patients' anxiety and expectations for the treatment				
1.5 Perform the complete physical examination				
1.6 Choose proper medical examinations items				
1.7 Application of basic diagnostic procedures skillfully				
1.8 Report the problems met in clinical work to the senior doctor, and analysis the problems				
1.9 Report clinical diagnosis and treatment plan to senior doctors according to specifications				
1.10 Application of evidence-based medicine principles, adopt proper diagnosis and treatment plan				
1.11 Make treatment plan considering patient’s gender, religious and education level				
1.12 Make decisions together with patients and their families				
1.13 Convey accurately the illness and advice for treatment to patients and their families in time				
**Factor IV: Interpersonal Communication**	60.60	0.952	59.75	0.947
3.1 Effective listening and ability to collect comprehensive information				
3.2 Effective communication skills				
3.3 Understand, trust, respect patient and their families				
3.4 Protect patients’ privacy				
3.5 Preserve patients’ right to know				
3.6 Application of ethical principles for patient care				
3.7 Comfort patients’ anger and misunderstanding				
3.8 Conflict resolution, management, and prevention				
3.9 Skillfully convey bad news to patients				
3.10 Respect patients’ diversity				
3.11 Have the skills to obtain the patient's informed consent				
**Factor V: Health promotion and disease prevention**	63.11	0.947	62.22	0.945
6.1 Prevention and control of infectious diseases, found infectious disease and report timely in the community				
6.2 Prevention of chronic non-communicable diseases				
6.3 Master of population health-related factors such as lifestyle, environment, and social etc.				
6.4 Understand the responsibilities to cooperate with the health system management				
6.5 Appropriate use of limited health care resources				
6.6 Familiar with social health insurance system				
6.7 Know about the impact of public health policies for population				
6.8 Participate in health promotion and disease prevention actively				
**Factor VI: Master of medical knowledge**	65.34	0.905	64.39	0.902
4.6 Agree with scientific standard and maintain the integrity of knowledge				
4.7 Keep updating medical knowledge and clinical skills				
4.8 Actively participate in continuing medical education				
4.9 Understand inadequacies of professional techniques and continuous study				
4.10 Know about self disadvantage and do self-improvement in practice				
4.11 Application of evidence-based medicine in clinical decision				
**Factor VII: Academic research**	67.00	0.915	65.94	0.918
8.1 Use critical thinking to deal with a variety of sources of information				
8.2 Have the ability to translate literature, spread and use knowledge				
8.3 Creative thinking and innovation ability				
8.4 Take part in science research actively				
8.5 Scientific research literature written and publish				
**Factor VIII: Teamwork**	68.41	0.932	67.47	0.929
5.5 Understand the principle of teamwork				
5.6 Be pleased to help colleague				
5.7 Understand the roles and responsibilities of others in the team				
5.8 Good coordination to avoid conflicts with team members				
5.9 Establish good cooperative relations with other departments				
5.10 Participate in other specialist team meetings				

### Confirmatory Factor Analysis

The full sample was used for CFA, which followed EFA to verify whether the hypothesized structure from EFA was consistent and valid. We adopted the most popular fit indices, including GFI, NFI, IFI, CFI, RMSEA, and SRMR. The results showed that all items had acceptable goodness of fit index. RMSEA and SRMR were less than 0.08 (RMSEA = 0.046, SRMR = 0.040), while GFI, NFI, IFI, and CFI were higher than 0.9 (GFI = 0.905, NFI = 0.903, IFI = 0.909, CFI = 0.909), leading to acceptable construct validity.

The values of construct reliability (CR) and the average variance extracted (AVE) were calculated for the convergent and discriminant validity. The CR value should be equal or higher than 0.70 to ensure the good convergence. And the value of AVE should exceed 0.5 to ensure the good discriminability [25]. As presented, all CR values of the factors were higher than 0.70, and all AVE values exceeded 0.50. Thus, we considered the convergent and discriminant validity acceptable (Table 6).

**Table 6:** The values of CR and AVE extracted for 8 factors.

	F1	F2	F3	F4	F5	F6	F7	F8
CR	0.954	0.945	0.933	0.948	0.947	0.901	0.919	0.933
AVE	0.599	0.569	0.518	0.623	0.691	0.646	0.694	0.698

## Discussion

Nowadays, medical education is moving to the third generation of revolution, which is called competency-based medical education [26–28]. The competency model is an efficient tool which is designed to evaluate the physicians’ qualification, and it will become a significant instrument for the medical education development in the future. This study systematically constructed physicians’ competency model containing 8 competence factors and 76 items in China. It is prerequisite to carefully verify the reliability and validity of the final scale. The indicators for CFA all reached the ideal value, which indicated good reliability and validity for the scale we constructed. Especially, we conducted a replication procedure for the EFA before embarking on the CFA. The replication analysis in EFA will allow researchers to provide readers information about the extent to which their EFA model meets these reasonable expectations for replicability [29,30].

Factor I appeared to focus upon the competency of management, which including information management, scheduling ability, project management and the management of medical charge, etc. Information management is the core competency of comprehensive quality for clinical physicians, and will become important quality for clinical medical treatment and medical scientific research. Excellent clinicians have high-ability time management, developing a scientific and health way to accomplish efficient work mode. Importantly, the clinicians should control the patient’s medical expenses as far as possible, to protect the right of patients and save the medical resources. Factor II, professionalism, included 13 items. The items addressed that clinical physicians should regard serving for people’s health as the highest standards of ethics. In the medical career, clinicians need to have the ultimate faith, including altruism, pursuit of excellence, sincere and trustworthy, strong sense of responsibility, and indifferent to fame and wealth [31].

13 items loaded on Factor III. This factor revealed a competent physician should make reasonable diagnosis and treatment plan, according to the integrated use of clinical knowledge, comprehensive and focused examination, necessary laboratory and equipment inspection. Factor IV, interpersonal communication, included 11 items. The communication between clinicians and patients included disease situation, diagnosis and treatment scheme, informed consent, right and obligation, and medical expenses [32]. The clinicians should give full consideration to the ideas of patients, and answer their questions timely.

Factor V, health promotion and disease prevention, which contained 8 items, emphasizing the importance of prevention which enable patients to eliminate or reduce health risk factors, as well as an ability to enhance well-being by providing health knowledge and behavior intervention. Health education is an effective measure to improve the quality of residents’ health, which guiding people to establish a scientific, civilized and healthy lifestyle [33]. Factor VI, master of medical knowledge, included 6 items. This factor focused on the significance of medical knowledge and lifelong learning ability. The progress of science and technology greatly changed the way of human learning, life and communication style, also promoting the medical education to a new stage of development [28]. In the 21st century, the medical workers face increasingly complex social and working environment, disposable medical education cannot meet the needs of clinicians to update knowledge, and lifelong learning is the inevitable trend of social development.

Factor VII, academic research, included 5 items. Medical research is a scientific way to explore medical knowledge, so as to improve physicians’ practical and creative thinking ability in the process of discovering, analyzing and dealing with problems comprehensively. Similarly, the research paper is an important part of medical research, which plays a vital role in promoting the development of medical science. Writing medical paper is one of the basic skills of medical science and technology workers must have. Factor VIII, teamwork, included 6 items. The items addressed the medical process was a team activity, which is the ability of comprehensive application for each member of the team. It is effective to make the patient’s diagnosis and treatment plan in the form of teamwork: promoting team cooperation ability, enhancing patient safety, improving the medical and nursing quality, increasing job satisfaction and maintaining the stability of the medical team [34].

Comparing the results of this study to those conducted in other countries, it is apparent that the competency model was different, which our study are in conformity with China's actual conditions. Different geographical features, customs, and historical backgrounds cause the cultural differences between China and other countries. Some research issues began to study the importance of cultural competence in other countries [35–38]. The highlight of the study is that the number of participants far exceeded the previous studies. Unlike previous studies conducted in China, the sample for this study included seven administrative regions, and the broad participation from a national sample adds to the credibility of the results.

However, there are several limitations in our study. Firstly, the questionnaire of competency model was constructed by clinicians, ignoring the opinions of others, such as nurses, patients and administrators. Secondly, due to the different competency requirement, the primary care physicians and physicians with different specialties or clinical settings were not covered in our study. Nonetheless, our study is a competency framework, which providing the theoretical and methodological basis for primary physicians and physicians with different specialties or clinical settings in China. Similarly, ACGME identified the competency framework applicable to most physicians, and the indicator system to evaluate these competencies firstly. On the basis of this competency framework, ACGME developed a list of physician competency models for different professions. Therefore, the subsequent studies should focus on constructing the physician competencies for primary care and different specialties or clinical settings in China. In addition, constructing and analyzing the competency model for different professional physicians will build and enrich the model of physician’s competency framework.

In conclusion, the competency model of clinicians has several practical implications. The results could be used as a measure to determine content for assessments of competency. The hospital administrators can adopt rapid adjustment measures to ensure clinical practice in accordance with the development of virtuous cycle. The evaluation results can be served as an important theoretical basis for the health administrative department, so as to promote the reform of clinical training way and content. Furthermore, the competency model can provide important reference basis for constructing physician competencies with different specialties or clinical settings. In educational settings, the tool could be used by students to self-evaluate their skill level both before and after learning activities. This feedback may help faculty members determine how their students perform.

## Conclusion

The instrument was shown to be both valid and reliable for measuring clinical physicians’ competency. Along with the deepening and refinement of standardized training, the overall level of clinicians will be increased in China, so as to cultivate clinical physicians with strong clinical practice, innovation and independent scientific research ability. 8 dimensions of core competencies were identified: information and management; professionalism; clinical skills and patient care; interpersonal communication; health promotion and disease prevention; master and use of medical knowledge; academic research; and teamwork.

## Supporting Information

S1 File This is the questionnaire described in manuscript. (PDF)

S1 Table Relevant data underlying the findings described in manuscript. (XLSX)

## Abbreviations

ACGME: Accreditation Council for Graduate Medical Education
AVE: average variance extracted
CanMEDS: Canadian Medical Education Directives for Specialists
CFA: confirmatory factor analysis
CFI: comparative fit index
CR: construct reliability
EFA: exploratory factor analysis
GFI: goodness of fit index
GMC: General Medical Council
GMP: Good Medical Practice
IFI: incremental fit index
KMO: Kaiser Meyer Olkin
NFI: normal fit index
RMSEA: root mean square error of approximation
SRMR: standardized root mean square residual
