# Supplementary material for: A Competency Model for Clinical Physicians in China: A Cross-Sectional Survey
Source: PLoS One. 2016 Dec 9;11(12):e0166252. doi: 10.1371/journal.pone.0166252 (PMC5147829; doi:10.1371/journal.pone.0166252)
Supplement: S1 File — (PDF) [file pone.0166252.s001.pdf]

Questionnaire on Competency of Chinese Clinicians

Introduction:

Competency is the broad base of qualified physicians, including professional knowledge, skills, attitudes and behavior, from the beginning of medical education in the school training. These results are of great significance to the setting of reference standards for national physician examinations and the guidance of undergraduate medical education and post - graduation education reform.

Information:

Sex ____ Age_____

Major ____________ Profession______________

Title____________ Post_________

Work Unit_____________________ （Hospital level： __ ）

Resident standardized training base □Yes □No Department_________

Unit address ______Province ______City ______County ______Street

Unit level □Province □City □County □Street

Highest degree □Bachelor □Master □Doctor

Work time： □Below 5 years □5 to 9 years □10 to 14 years □15 to 19 years □20 years and above

Categories’ importance:

1-definitely not important; 2-not important; 3-neural; 4-important; 5-definitely important

Items	Competency importance				
	1	2	3	4	5
**Clinical skills and patient care**					
1.1 Prudent practice, pay attention to patients' safety	○	○	○	○	○
1.2 Collect important medical histories	○	○	○	○	○
1.3 Complete medical documents according to specifications	○	○	○	○	○
1.4 Understand patients' anxiety and expectations for the treatment	○	○	○	○	○
1.5 Perform the complete physical examination	○	○	○	○	○
1.6 Choose proper medical examinations items	○	○	○	○	○
1.7 Application of basic diagnostic procedures skillfully	○	○	○	○	○
1.8 Report the problems met in clinical work to the senior doctor, and analysis the problems	○	○	○	○	○
1.9 Report clinical diagnosis and treatment plan to senior doctors according to specifications	○	○	○	○	○
1.10 Application of evidence-based medicine principles, adopt proper diagnosis and treatment plan	○	○	○	○	○
1.11 Make treatment plan considering patient’s gender, religious and education level	○	○	○	○	○
1.12 Make decisions together with patients and their families	○	○	○	○	○
1.13 Convey accurately the illness and advice for treatment to patients and their families in time	○	○	○	○	○
1.14 Do a good job in clinical consultation and referral	○	○	○	○	○
1.15 Identify and be able to carry out emergency rescue of patients with acute, severe and dangerous	○	○	○	○	○
1.16 Proper hospice care for dying patients	○	○	○	○	○
1.17 Strict implementation of medical management-related laws, regulations and treatment technical specifications	○	○	○	○	○
1.18 Report and analyze medical errors as required	○	○	○	○	○
1.19 Reduce the excessive use of medical resources	○	○	○	○	○
**Professionalism**					
2.1 Adhere to heal the sick, serving the people wholeheartedly	○	○	○	○	○
2.2 Love one’s own career	○	○	○	○	○
2.3 Responsibility	○	○	○	○	○
2.4 Sincere and trustworthy	○	○	○	○	○
2.5 Self-regulation	○	○	○	○	○
2.6 Sympathy to patients	○	○	○	○	○

2.7 Patients first, maintaining patients' rights and interests	○ ○ ○ ○ ○
2.8 Fair and honest	○ ○ ○ ○ ○
2.9 Self protection awareness with legal	○ ○ ○ ○ ○
2.10 Precise and careful	○ ○ ○ ○ ○
2.11 Doctors should pay attention to their health	○ ○ ○ ○ ○
2.12 Patience and endurance	○ ○ ○ ○ ○
2.13 Emphasis on self-evaluation and peer-review	○ ○ ○ ○ ○
2.14 With a certain compressive capacity	○ ○ ○ ○ ○
2.15 With a certain response to emergency response capabilities	○ ○ ○ ○ ○
2.16 Altruism	○ ○ ○ ○ ○
2.17 Pursuit of excellence	○ ○ ○ ○ ○
2.18 Indifferent to fame and fortune	○ ○ ○ ○ ○
2.19 With keen insight	○ ○ ○ ○ ○
2.20 Recognize and eliminate any profit related activities	○ ○ ○ ○ ○
2.21 Actively participate in internal review and external inspection	○ ○ ○ ○ ○
**Interpersonal Communication**	
3.1 Effective listening and ability to collect comprehensive information	○ ○ ○ ○ ○
3.2 Effective communication skills	○ ○ ○ ○ ○
3.3 Understand, trust, respect patient and their families	○ ○ ○ ○ ○
3.4 Protect patients' privacy	○ ○ ○ ○ ○
3.5 Preserve patients' right to know	○ ○ ○ ○ ○
3.6 Application of ethical principles for patient care	○ ○ ○ ○ ○
3.7 Comfort patients' anger and misunderstanding	○ ○ ○ ○ ○
3.8 Conflict resolution, management, and prevention	○ ○ ○ ○ ○
3.9 Skillfully convey bad news to patients	○ ○ ○ ○ ○
3.10 Respect patients' diversity	○ ○ ○ ○ ○
3.11 Have the skills to obtain the patient's informed consent	○ ○ ○ ○ ○
3.12 Encourage patients to discuss, ask questions, and communicate with each other	○ ○ ○ ○ ○
3.13 Have a certain ability to speak	○ ○ ○ ○ ○
**Master of medical knowledge**	
4.1 Master the necessary physical and chemical knowledge	○ ○ ○ ○ ○

4.2 Master the biomedical knowledge	○ ○ ○ ○ ○
4.3 Master the clinical medical knowledge	○ ○ ○ ○ ○
4.4 Familiar with preventive medicine knowledge	○ ○ ○ ○ ○
4.5 Familiar with the theories of the humanities and social sciences	○ ○ ○ ○ ○
4.6 Agree with scientific standard and maintain the integrity of knowledge	○ ○ ○ ○ ○
4.7 Keep updating medical knowledge and clinical skills	○ ○ ○ ○ ○
4.8 Actively participate in continuing medical education	○ ○ ○ ○ ○
4.9 Understand inadequacies of professional techniques and continuous study	○ ○ ○ ○ ○
4.10 Know about self disadvantage and do self-improvement in practice	○ ○ ○ ○ ○
4.11 Application of evidence-based medicine in clinical decision	○ ○ ○ ○ ○
**Teamwork**	
5.1 Respect their superiors	○ ○ ○ ○ ○
5.2 Respect their subordinates	○ ○ ○ ○ ○
5.3 Obey the organization and management	○ ○ ○ ○ ○
5.4 Develop a patient-managed treatment plan in a team-based manner	○ ○ ○ ○ ○
5.5 Understand the principle of teamwork	○ ○ ○ ○ ○
5.6 Be pleased to help colleague	○ ○ ○ ○ ○
5.7 Understand the roles and responsibilities of others in the team	○ ○ ○ ○ ○
5.8 Good coordination to avoid conflicts with team members	○ ○ ○ ○ ○
5.9 Establish good cooperative relations with other departments	○ ○ ○ ○ ○
5.5 Understand the principle of teamwork	○ ○ ○ ○ ○
**Health promotion and disease prevention**	
6.1 Prevention and control of infectious diseases, found infectious disease and report timely in the community	○ ○ ○ ○ ○
6.2 Prevention of chronic non-communicable diseases	○ ○ ○ ○ ○
6.3 Master of population health-related factors such as lifestyle, environment, and social etc.	○ ○ ○ ○ ○
6.4 Understand the responsibilities to cooperate with the health system management	○ ○ ○ ○ ○

6.5 Appropriate use of limited health care resources	○ ○ ○ ○ ○
6.6 Familiar with social health insurance system	○ ○ ○ ○ ○
6.7 Know about the impact of public health policies for population	○ ○ ○ ○ ○
6.8 Participate in health promotion and disease prevention actively	○ ○ ○ ○ ○
6.9 Understand the interaction and impact of health care activities with the health system and society as a whole	○ ○ ○ ○ ○
6.10 Understand international health status and global health	○ ○ ○ ○ ○
**Information and management**	
7.1 Search and analyze medical information from different databases	○ ○ ○ ○ ○
7.2 Search and analyze medical information from different databases	○ ○ ○ ○ ○
7.3 Reasonable control of the patient's medical expenses	○ ○ ○ ○ ○
7.4 Maintain complete medical record	○ ○ ○ ○ ○
7.5 Effectively plan the work and career	○ ○ ○ ○ ○
7.6 Appropriate use of time, plan to handle own activities	○ ○ ○ ○ ○
7.7 Management capabilities, including patient management, internship student management	○ ○ ○ ○ ○
7.8 Constantly improve management capacities of organization and coordination in practice	○ ○ ○ ○ ○
7.9 Adequately demonstrate leadership in the team	○ ○ ○ ○ ○
7.10 Master at least one foreign language	○ ○ ○ ○ ○
7.11 Computer skills	○ ○ ○ ○ ○
7.12 Provide guidance and teaching to colleague and medical students if necessary	○ ○ ○ ○ ○
7.13 Apply the knowledge and technology of pedagogic to faculty training	○ ○ ○ ○ ○
7.14 Evaluate the training object using advanced clinical assessment	○ ○ ○ ○ ○
**Academic research**	
8.1 Use critical thinking to deal with a variety of sources of information	○ ○ ○ ○ ○
8.2 Have the ability to translate literature, spread and use	○ ○ ○ ○ ○

knowledge	
8.3 Creative thinking and innovation ability	○ ○ ○ ○ ○
8.4 Take part in science research actively	○ ○ ○ ○ ○
8.5 Scientific research literature written and publish	○ ○ ○ ○ ○
